# Individual and regional factors associated with suicidal ideation among Korean elderly: a multilevel analysis of the Korea Community Health Survey

**DOI:** 10.4178/epih.e2019022

**Published:** 2019-05-26

**Authors:** Sang Hee Jeong, Byung Chul Chun

**Affiliations:** 1 Graduate School of Nursing, Konkuk University, Chungju, Korea; 2 Department of Preventive Medicine, Korea University College of Medicine, Seoul, Korea; 3 Department of Epidemiology and Health Informatics, Graduate School of Public Health, Korea University, Seoul, Korea

**Keywords:** Suicidal ideation, Aged, Multilevel analysis, Socioeconomic factors, Korea Community Health Survey

## Abstract

**OBJECTIVES:**

This study aimed to identify the individual and regional characteristics that influence suicidal ideation among the Korean elderly population.

**METHODS:**

Using data collected from the 2013 Korea Community Health Survey, a multilevel analysis was performed to establish an understanding of individual behavioral patterns and regional influences on suicidal ideation.

**RESULTS:**

Among the 77,407 individuals sampled, 11,236 (14.5%) elderly people over 60 years of age experienced suicidal ideation. Among individual factors, age, frequency of communication with friends, religious activity, social activity, leisure activity, trust in neighbors, subjective stress level, depressive symptoms, and subjective health status were significantly associated with suicidal ideation. The results showed that the lower the regional deprivation level, the higher the suicidal ideation odds ratio. In terms of regional size, the most significant effects were found in rural areas.

**CONCLUSIONS:**

This study suggested that suicidal ideation in the elderly is associated with community factors, such as the regional deprivation index, as well as personal factors.

## INTRODUCTION

It has been argued that elderly persons in modern society may exhibit extreme and negative behaviors such as suicide if they fail to adequately deal with complex problems, such as extended life expectancy, loss of social status due to the development of nuclear families, weakening of interpersonal relationships, economic instability, feelings of loneliness and helplessness, and lack of community integration [1,2].

In addition, low education levels, absence of social activity, high levels of depressive symptoms, poor subjective health status, high stress levels [3-5], and low social support [6] among individuals living alone have been reported as causes of suicidal ideation. Previous studies [3,5] have often identified the causes of suicide by focusing on mental-health problems among elderly persons living alone and have presented the causal characteristics of suicidal ideation among elderly persons in terms of individual and social aspects. Further, studies comparing individual factors and regional factors, including socioeconomic factors, among elderly persons are rare and few domestic studies have performed a multilevel analysis of regional factors as well as individual characteristics [7,8].

Factors affecting individuals’ health are a combination of personal characteristics and regional factors [9]. In this study, the social and physical environment of an administrative district is used to represent the characteristics of a “region” [10]. Individual and regional factors influencing suicidal ideation can be identified through a multilevel analysis, an estimation method for multilevel data attributes in which observational data units are embedded in a higher-level group which can determine whether the resulting estimates are statistically significant [11]. This study aimed to investigate individual and regional factors affecting suicidal ideation in individuals aged 60 years or older.

## MATERIALS AND METHODS

### Study subjects

Although the Korean Elderly Welfare Act stipulates that individuals aged 65 or older are elderly persons, the National Pension Act stipulates that individuals are eligible for an old-age pension at age 60, and individuals can also use elderly-related welfare facilities at age 60. In this study, the elderly age group was defined as consisting of individuals aged 60 years or older, considering their community activities and economic profile. Of 228,781 respondents in the 2013 Korea Community Health Survey (KCHS), 80,360 were selected, excluding those under the age of 60. Of these, 77,407 subjects were finally selected for this study, excluding 2,954 with missing information who declined to respond or responded with “do not know” to 15 essential items (age, type of household, education level, frequency of communication with relatives/family, frequency of communication with friends, religious activity, fellowship activity, leisure activity, trust in neighbors, safety level, monthly household income level, economic activity status, depressive symptoms, subjective stress level, and subjective health level).

### Data

This study used raw data from the 2013 KCHS, which was administered to adults aged 19 years or older by 253 community health centers (an average sample size of 900 per community center) in cities, *gun* (counties), and gu (districts) [12]. These data were used because the “Act for the Prevention of Suicide and the Creation of a Culture of Respect for Life” was implemented in 2012, and the Ministry of Health and Welfare Affairs (MOHWA) establishes a National Suicide Prevention Master Plan every five years. Therefore, this study intended to investigate factors influencing suicidal ideation among elderly persons in 2013, shortly after the Act for the Prevention of Suicide and Creation of a Culture of Respect for Life was implemented and the 2nd National Suicide Prevention Mater Plan (2009-2013) was terminated.

Suicidal ideation, a dependent variable, was classified according to the presence or absence of suicidal ideation during the past year, and those who responded with “yes” were classified as the suicidal ideation group. Independent variables included age (individuals in their 60s, 70s, and 80s or older), type of household (elderly person living along; other households: elderly person living with non-relatives, elderly person living with relatives; elderly person living with spouse; elderly person living with children), education level (no formal education, elementary-school graduate, junior high-school graduate, high-school graduate, university or higher graduate). Other independent variables included frequency of communication with relatives/family (less than once a month, 1-3 times a month, 1-3 times a week, and 4 times a week or more); Religion, social and leisure activities (at least once a month); satisfaction or dissatisfaction with trust in neighbors and safety level; monthly household income (Korean won <2.00 million, 2.00-2.99 million, 3.00-3.99 million, 4.00-4.99 million, and 5.00 million or more); economic activity (at least one hour per week of incomegenerating activities, or at least 18 hours of unpaid housework); the presence of absence of depressive symptoms (for more than two weeks during the past year); subjective stress level (very high, high, low, very low); and subjective health level (good, poor). These 15 essential items constitute the individual variables.

Regarding the regional population variable, 69 metropolitan cities were classified as large cities, 101 cities as small-medium cities, and 83 counties (gun) into rural areas, reflecting the 253 administrative districts in 2013. The regional variables were considered to be regional characteristics that could influence suicidal ideation differently among individuals residing in urban and rural areas [13]. These areas were classified by population size into three groups (large cities, small-medium cities and rural areas) according to existing administrative districts to minimize the effects of confounding factors in addition to regional factors [8].

Regarding changes made to administrative districts, Yeoju-gun was upgraded to Yeoju City in 2013, and its name was changed accordingly. In addition, Yeongi-gun was integrated into Sejong Metropolitan Autonomous City in 2012, which was reflected in the analysis. The regional deprivation index is a major index used to determine disparities in regional health and quality of life and was scored by calculating each normalized z-score for nine regional characteristics such as lack of car ownership (classified within urban and rural areas), poor housing conditions, the percentage of householders living alone, the percentage of female householders, apartment ownership (as a percentage of all households), the percentage of the population with a high-school education or below among those aged 35-64 (lower education level), lower socioeconomic level, and the percentage of the population that is elderly (aged 65 or older) from the 2010 Population Census data [14]. The financial autonomy ratio was calculated based on independent revenue (local tax+non-tax revenue)/local government budget size×100, and is an indicator of the local government’s ability to spend independently [8,15]. Using the 2010 Population Census data, the national basic living allowance ratio was calculated based on the number of the national basic living allowance recipients in 2013 by multiplying the proportion of recipients within the total population in a given region by 100 and can predict absolute poverty levels among the total population within the region [7,8].

### Statistical analysis

Based on data from the 2013 KCHS, descriptive statistics and the χ^2^-test were used to analyze the individual characteristics of elderly persons according to the presence or absence of suicidal ideation. A multilevel analysis was performed to determine the relationship between individual behaviors and the likelihood of suicidal ideation among elderly persons and the effects of regional or group influences.

Three models were constructed for the multilevel analysis. Model 1 is a basic model, showing the regional differences in suicidal ideation that did not include individual and regional factors. Model 2 is an individual-level model that was used to determine the influence of individual-level characteristics on suicidal ideation. Model 3 is a group-level model for estimating regional influence on suicidal ideation that included individual and regional factors. The intraclass correlation coefficient (ICC) was used to investigate individual effects (%) and regional effects (%) in the overall distribution of suicidal ideation in elderly persons, and was calculated using Lee & Heo [16]’s formula as follows:

$$\mathrm{ICC}=\frac{\hat{\sigma_{1}^{2}}}{\hat{\sigma_{1}^{2}}+\pi^{2}/3}\;\sigma_{i}^{2}:\mathrm{residual}\;\mathrm{variance}\;\mathrm{between}\;\mathrm{communities}\;\pi^{2}/3:\mathrm{residual}\;\mathrm{variance}\;\mathrm{between}\;\mathrm{individuals}$$

Data analysis was performed using SAS version 9.4 (SAS Institute Inc., Cary, NC, USA). Statistical significance was set at α=0.05.

### Ethics statement

The study protocol was approved by the Institutional Review Board (IRB) of Korea University (no. KU-IRB-17-EX-92-A-1). Informed consent was confirmed (or waived) by the IRB.

## RESULTS

As shown in Table 1, 11,236 (14.5%) out of 77,407 respondents in the KCHS reported suicidal ideation during the past year. Suicidal ideation rates were higher among older respondents; those whose communicated with friends less than once a month (20.4%); those with no religion (15.2%), social (19.2%), and leisure activity (15.6%); those who were dissatisfied with their trust in neighbors (19.5%); those with depressive symptoms (61.8%); those with higher subjective stress levels (23.3%); and those with poor subjective health levels (23.3%).

Table 2 shows regional socioeconomic characteristics. The regional deprivation index ranged between a minimum deprivation status of -17.7 and a maximum deprivation status of 17.0, and the mean value was 0. The mean basic living allowance ratio among regions was 3.7%, ranging from 0.65 in the region with the lowest ratio to 9.6% in the region with the highest. The mean financial autonomy ratio was 46.4%, ranging from 7.3% in the region with the lowest financial autonomy to 90.0% in the region with the highest.

Table 3 presents the results of the multilevel analysis model that considered individual and regional factors influencing suicidal ideation among elderly subjects. In model 1, which investigated regional differences in suicidal ideation but excluded the relationships between independent variables, the ICC value was calculated as 0.066. This implies that the magnitude of the variance that occurred when residing in a region accounted for 6.6% of the variance in the overall suicidal ideation rate.

The results of model 2 reflecting individual factors showed that higher age, living alone, and communication with friends less than once a month were associated with a higher likelihood of suicidal ideation. In addition, the absence of religion, fellowship, and leisure activities and dissatisfaction with trust in neighbors were shown to be associated with a higher odds ratio (OR) for suicidal ideation. The presence of depressive symptoms, higher subjective stress levels, and poor subjective health levels were also found to be associated with a higher likelihood of experiencing suicidal ideation. In model 2, the ICC value was calculated as 7.8%, which is interpreted as the percentage of individual factor variance within the total variance observed in the likelihood of suicidal ideation among the elderly subjects.

Model 3 confirmed the influence of regional factors on suicidal ideation when controlling for the individual factors, and the results of analyzing the regional factors showed that the basic living allowance ratio had a positive effect on suicidal ideation among the elderly subjects, but it was not statistically significant. The financial autonomy variable also showed the same result. When the regional deprivation index was increased by one unit, the probability of suicidal ideation in elderly subjects increased 0.98 times (95% confidence interval [CI], 0.96 to 0.99). Regarding area population size, the OR for suicidal ideation was 1.44 times higher in rural areas than in large cities (95% CI, 0.98 to 2.10). In model 3, the variance of regional factors accounted for 7.7% of the total variance in the likelihood of suicidal ideation among elderly subjects.

## DISCUSSION

Regarding the individual factor of age, a higher age was associated with a higher likelihood of suicidal ideation [17]. Because of population aging and increasing life expectancy, the life cycle of elderly persons within particular age groups is clearly different from that in the past, it is highly useful to examine suicidal ideation among elderly persons by age group. This study’s results found that only the frequency of communication with friends had a significant influence on the likelihood of experiencing suicidal ideation, suggesting that communication with friends played a greater role in emotional support than did communication with relatives/family [18]. Therefore, it can be inferred that elderly persons’ interactions with peers possessing similar characteristics allows them to experience empathy or sympathy.

A study by Lee [19] claimed that social activity was a major mediator in the relationship between social isolation and suicidal ideation and can inhibit the effects of social isolation on suicidal ideation. In this study, it was also suggested that social activities such as religious activities, fellowship, and leisure activities acted as controlling factors. A study by Noguchi et al. [17] found that elderly people living in trustworthy communities were less likely to experience suicidal ideation with psychological distress, regardless of self-perception. This study also showed the same result in that forming bonds with others through a sense of trust in interpersonal relationship positively affected mental health. However, future data on social networks that can carefully measure the influence of the strength and depth of relationships with neighbors are required. The results of this study showed that the presence of depressive symptoms, higher subjective stress levels, and poorer subjective health levels were associated with a higher likelihood of suicidal ideation, which was consistent with the results of previous studies [17,20,21]. These factors have a direct influence on suicidal ideation, and are seen as important individual factors, especially for policies related to suicide prevention.

Therefore, in examining the likelihood of suicidal ideation according to elderly persons’ individual factors, it was confirmed that such individual factors influencing suicidal ideation are in fact influenced by each other; their impact is influenced by their interactive effects.

Based on the findings of a previous study [8], this study examined the regional deprivation index, financial autonomy, and basic living allowance ratio, which reflect socioeconomic characteristics, and used the values as regional factors in the multilevel analysis. To estimate the validity of such a multilevel analysis, acceptable ICC values are commonly accepted as 5-25% in social science research. If the ICC value is less than 5%, a single-level model is considered appropriate [22]. The ICC value in model 3 was 7.7%, which indicates the fit of the model for multilevel analysis. The ICC value (7.8%) in model 2 is slightly higher than in model 1 (6.6%), and similar results have been reported in other studies that performed multilevel analyses using domestic data [23-25]. However, these studies did not address the causes of the increased ICC, and further studies are thus needed to explain this increase. Furthermore, in model 3, which considers regional factors, ICC was very slightly lower (0.001) than in model 2. The regional deprivation index, considered as a regional factor, was statistically significant and could possibly be interpreted as a valid regional factor. However, it was judged that the regional factor (or the regional deprivation index) contributed a little in explaining the overall variables in this model. It was difficult to determine whether this was due to the characteristics of regional data in Korea (such as the fact that available data regarding regional characteristics do not meticulously reflect each individuals’ living environment or regional characteristics because the scope of the data is large, by city, gun, and gu), or other factors such as ecological errors. Further studies regarding this are needed.

A study by Skapinakis et al. [26] claimed that the regional deprivation index was positively associated with mental health. However, a study by Shin & Shin [8] showed contradictory results and indicated that the difference between the studies was related to rural characteristics that were difficult to explain. In particular, the effects of individual variables that unaccounted for were seen in the results as the influence of social support and structural regional factors. Model 3 in this present study also showed similar results.

The regional deprivation index is a continuous variable and has an absolute value, but it is necessary to appropriately categorize and analyze it in the future to interpret the ORs.

In this study, variables such as basic living allowance ratio and financial autonomy were also used to determine whether the outcome of the deprivation index variable were related to the attributes of the variables themselves or with the mediating effect of other variables. The results showed that the deprivation index still operated in the reverse direction, similar to the results of model 3, and that the basic living allowance ratio and financial autonomy were not statistically significant. However, the results of model 3 showed that the OR of suicidal ideation in rural areas was 1.44 times higher than that in large cities, but the significance level was close to 5% (p=0.061), and the CI included the value of 1 (95% CI, 0.98 to 2.10). Therefore, it is necessary to interpret this as indicating a range of influence on suicidal ideation considering the approximate CI, rather than understanding it as a limited result as interpreted by statistical test criteria.

In light of the fact that the suicide-related questions contained in the KCHS are very sensitive, and responses to them may differ, these questions consist of a minimum number that suited for use in communities [27] and are based on the suicidal ideation-related questionnaire by Bertolote et al. [28]. Additionally, the KCHS is conducted every year with selected 4-year rotating survey items and has been used to establish community health care in accordance with the Community Health Act [29]. This suggests that the suicide-related items contained in the 2013 KCHS could also serve as sufficient data to reflect the demand for community mental health and as index data, particularly in the healthcare industry.

This study has several limitations as follows. First, the items on suicidal ideation contained in the KCHS provided only limited information because of the survey’s rotating system and categorical form. To overcome these problems, items must be constructed that are suited for suicide prevention at the national level and that can invigorate the annual data. Second, unlike measurements of population size in other countries, Korea does not have clear boundaries between the residence and the sphere of activities of residents in communities. Therefore, objective data or pilot studies are needed to classify regions into comparable areas nationwide, such as living areas and communities rather than administrative districts.

In conclusion, this study suggests that factors affecting suicidal ideation among elderly people over age 60 include not only individual factors but also regional factors such as deprivation index.

## Figures and Tables

**Table 1.** Socio-demographic characteristics of the study subjects and suicidal ideation by group

Variables	Categories	Suicidal ideation		
		Yes	No	p-value
Age (yr)	60-69	3,927 (11.3)	30,927 (88.7)	<0.001
	70-79	5,196 (16.1)	27,119 (83.9)	
	≥80	2,113 (20.6)	8,125 (79.4)	
Types of households	Single	3,248 (20.7)	12,399 (79.3)	<0.001
	Living with spouse	2,536 (13.6)	16,090 (86.4)	
	Living with children	886 (16.7)	4,427 (83.3)	
	Other	4,566 (12.1)	33,255 (87.9)	
Education (graduation)	No formal education	3,717 (21.9)	13,243 (78.1)	<0.001
	Elementary school	4,780 (15.0)	27,174 (85.0)	
	Middle school	1,290 (10.8)	10,713 (89.2)	
	High school	1,056 (9.4)	10,141 (90.6)	
	College or more	393 (7.4)	4,900 (92.6)	
Frequency of communication with relatives (family)	Less than once a month	2,175 (17.7)	10,131 (82.3)	<0.001
	1-3 times a month	2,993 (14.0)	18,321 (86.0)	
	1-3 times a week	3,537 (13.9)	21,912 (86.1)	
	4 times a week or more	2,531 (13.8)	15,807 (86.2)	
Frequency of communication with friends	Less than once a month	5,078 (20.4)	19,831 (79.6)	<0.001
	1-3 times a month	2,030 (11.3)	15,991 (88.7)	
	1-3 times a week	1,841 (11.4)	14,382 (88.6)	
	4 times a week or more	2,287 (12.5)	15,967 (87.5)	
Religious activity	Yes	3,140 (13.0)	20,973 (87.0)	<0.001
	No	8,096 (15.2)	45,198 (84.8)	
Fellowship activity	Yes	4,065 (10.2)	35,922 (89.8)	<0.001
	No	7,171 (19.2)	30,249 (80.8)	
Leisure activity	Yes	731 (7.2)	9,471 (92.8)	<0.001
	No	10,505 (15.6)	56,700 (84.4)	
Trust in neighbors	Satisfactory	8,521 (13.4)	54,973 (86.6)	<0.001
	Dissatisfactory	2,715 (19.5)	11,198 (80.5)	
Safety level	Satisfactory	9,229 (13.8)	57,866 (86.2)	<0.001
	Dissatisfactory	2,007 (19.5)	8,305 (80.5)	
Monthly household income level (10^4^ Korean won)	<200	8,945 (16.6)	44,823 (83.4)	<0.001
	200-299	1,030 (10.4)	8,847 (89.6)	
	300-399	514 (9.2)	5,074 (90.8)	
	400-499	305 (9.7)	2,851 (90.3)	
	≥500	442 (8.8)	4,576 (91.2)	
Presence of economic activity	Yes	3,663 (10.7)	30,516 (89.3)	<0.001
	No	7,573 (17.5)	35,655 (82.5)	
Depressive symptoms	Yes	3,169 (61.8)	1,958 (38.2)	<0.001
	No	8067 (11.2)	64,213 (88.8)	
Subjective stress level	Very high	1,239 (56.5)	954 (43.5)	<0.001
	High	4,723 (32.1)	9,982 (67.9)	
	Low	3,818 (10.9)	31,168 (89.1)	
	Very low	1,456 (5.7)	24,067 (94.3)	
Subjective health level	Good	3,464 (7.9)	40,560 (92.1)	<0.001
	Poor	7,772 (23.3)	25,611 (76.7)	

Values are presented as number (%).

**Table 2.** Descriptive statistics of regional characteristics

Regional characteristics	Mean±SD	Min	Max
Deprivation index	0.0±7.8	-17.7	17.0
Basic living allowance ratio (%)	3.7±1.8	0.65	9.6
Financial autonomy (%)	46.4±24.3	7.3	90.0

SD, standard deviation; Min, minimum; Max, maximum.

**Table 3.** Results of multilevel analysis of suicidal ideation considering both individual and community factors

Factors	Model 1	Model 2	Model 3		
			p-value	OR (95% CI)	p-value
Age group (yr)					
60-69	-	1.00 (reference)	-	1.00 (reference)	-
70-79	-	1.12 (1.06, 1.18)	<0.001	1.12 (1.06, 1.18)	<0.001
≥80	-	1.46 (1.35, 1.58)	<0.001	1.46 (1.35, 1.57)	<0.001
Types of households	-				
Single	-	1.19 (1.08, 1.31)	<0.001	1.19 (1.08, 1.31)	<0.001
Other	-	0.85 (0.78, 0.94)	0.001	0.85 (0.78, 0.94)	0.001
Living with spouse	-	0.96 (0.87, 1.06)	0.440	0.96 (0.87, 1.06)	0.450
Living with children	-	1.00 (reference)	-	1.00 (reference)	-
Education (graduation)					
No formal education	-	1.56 (1.36, 1.78)	<0.001	1.56 (1.37, 1.78)	<0.001
Elementary School	-	1.28 (1.13, 1.45)	<0.001	1.28 (1.13, 1.46)	<0.001
Middle school	-	1.10 (0.96, 1.25)	0.190	1.10 (0.96, 1.26)	0.180
High school	-	1.06 (0.92, 1.21)	0.440	1.06 (0.92, 1.21)	0.430
College or more	-	1.00 (reference)	-	1.00 (reference)	-
Frequency of communication with relatives (family)					
Less than once a month	-	1.01 (0.94, 1.09)	0.780	1.01 (0.94, 1.09)	0.800
1-3 times a month	-	1.01 (0.94, 1.08)	0.780	1.01 (0.94, 1.08)	0.790
1-3 times a week	-	1.04 (0.97, 1.11)	0.270	1.04 (0.97, 1.11)	0.270
4 times a week or more	-	1.00 (reference)	-	1.00 (reference)	-
Frequency of communication with friends					
Less than once a month	-	1.18 (1.10, 1.26)	<0.001	1.18 (1.10, 1.26)	<0.001
1-3 times a month	-	0.97 (0.90, 1.05)	0.450	0.97 (0.90, 1.05)	0.440
1-3 times a week	-	0.93 (0.87, 1.01)	0.077	0.93 (0.87, 1.01)	0.075
4 times a week or more	-	1.00 (reference)	-	1.00 (reference)	-
Religious activity					
Yes	-	1.00 (reference)	-	1.00 (reference)	-
No	-	1.14 (1.09, 1.21)	<0.001	1.15 (1.09, 1.21)	<0.001
Fellowship activity					
Yes	-	1.00 (reference)	-	1.00 (reference)	-
No	-	1.22 (1.16, 1.29)	<0.001	1.22 (1.16, 1.29)	<0.001
Leisure activity					
Yes	-	1.00 (reference)	-	1.00 (reference)	-
No	-	1.22 (1.11, 1.34)	<0.001	1.22 (1.11, 1.34)	<0.001
Trust in neighbors					
Satisfactory	-	1.00 (reference)	-	1.00 (reference)	-
Dissatisfactory	-	1.27 (1.19, 1.35)	<0.001	1.27 (1.19, 1.35)	<0.001
Safety level					
Satisfactory	-	1.00 (reference)	-	1.00 (reference)	-
Dissatisfactory	-	1.18 (1.11, 1.27)	<0.001	1.19 (1.11, 1.27)	<0.001
Monthly household income level (104 KRW)					
<200	-	1.36 (1.21, 1.54)	<0.001	1.36 (1.21, 1.54)	<0.001
200-299	-	1.17 (1.02, 1.33)	0.023	1.17 (1.02, 1.33)	0.022
300-399	-	1.00 (0.86, 1.17)	0.950	1.01 (0.87, 1.17)	0.950
400-499	-	1.09 (0.92, 1.30)	0.310	1.09 (0.92, 1.30)	0.310
≥500	-	1.00 (reference)	-	1.00 (reference)	-
Presence of economic activity					
Yes	-	1.00 (reference)	-	1.00 (reference)	-
No	-	1.26 (1.19, 1.33)	<0.001	1.26 (1.19, 1.33)	<0.001
Depressive symptoms					
Yes	-	6.74 (6.28, 7.23)	<0.001	6.74 (6.28, 7.23)	<0.001
No	-	1.00 (reference)	-	10.06 (8.96, 11.29)	-
Subjective stress level					
Very high	-	10.05 (8.95, 11.28)	<0.001		<0.001
High	-	5.54 (5.16, 5.94)	<0.001	5.54 (5.17, 5.95)	<0.001
Low	-	2.09 (1.96, 2.24)	<0.001	2.09 (1.96, 2.24)	<0.001
Very low	-	1.00 (reference)	-	1.00 (reference)	-
Subjective health level					
Good	-	1.00 (reference)	-	1.00 (reference)	-
Poor	-	1.88 (1.79, 1.98)	<0.001	1.88 (1.79, 1.98)	<0.001
Area population size					
Small-medium city	-	-	-	1.01 (0.80, 1.28)	0.920
Rural	-	-	-	1.44 (0.98, 2.10)	0.061
Large city	-	-	-	1.00 (reference)	-
Deprivation index	-	-	-	0.98 (0.96, 0.99)	0.018
Basic living allowance ratio	-	-	-	1.03 (0.96, 1.11)	0.400
Financial autonomy	-	-	-	1.00 (0.99, 1.01)	0.710
ICC	0.066	0.078		0.077	
Variances	0.231	0.281		0.276	
χ^2^	75,879.48	70,215.40		70,244.05	

Values are presented as odds ratio (95% confidence interval). KRW, Korean won; ICC, intraclass correlation coefficient.

## SUPPLEMENTARY MATERIALS

Korean version is available at http://www.e-epih.org/.
