# Supplementary material for: Individual and regional factors associated with suicidal ideation among Korean elderly: a multilevel analysis of the Korea Community Health Survey
Source: Epidemiol Health. 2019 May 26;41:e2019022. doi: 10.4178/epih.e2019022 (PMC6661470; doi:10.4178/epih.e2019022)
Supplement: Supplementary file 1 [file epih-41-e2019022-supplementary.pdf]

Volume: 41, Article ID: e2019022
https://doi.org/10.4178/epih.e2019022

Individual and regional factors associated with suicidal ideation among Korean elderly: a multilevel analysis of the Korea Community Health Survey

Sang Hee Jeong[1], Byung Chul Chun[2]

[1] Graduate School of Nursing, Konkuk University, Chungju

[2]Department of Preventive Medicine, Korea University Medical College; Department of Epidemiology and Health Informatics, Graduate School of Public Health, Korea University

Running Title: Multilevel analysis on suicidal ideation of the Korean elderly

Corresponding author: Byung Chul Chun, MD, PhD, MPH

Department of Preventive Medicine, Korea University Medical College

73 Inchon-ro, Seongbuk-gu, Seoul 02841, Korea

Phone:+82-2-2286-1169

e-mail: chun@korea.ac.kr

CONFLICT OF INTEREST

The authors have no conflicts of interest to declare for this study.

Volume: 41, Article ID: e2019022
https://doi.org/10.4178/epih.e2019022

Abstract

Objectives: This study aimed to identify the individual and regional characteristics that influence suicidal ideation among the Korean elderly population.

Methods: Using data collected from the 2013 Community Health Survey, a multilevel analysis was performed to establish an understanding of individual behavioral patterns and regional influences on suicidal ideation.

Results: Among the 77,407 individuals sampled, 11,236 (14.5%) elderly people over 60 years of age experienced suicidal ideation. Among individual factors, age, frequency of communication with friends, religious activity, social activity, leisure activity, trust in neighbors, subjective stress level, depressive mood, and subjective health status were significantly associated with suicidal ideation. The results showed that the lower the regional deprivation level, the higher the suicidal ideation odds ratio. In terms of regional size, the most significant effects were found in rural areas.

Conclusion: This study suggested that suicidal ideation in the elderly is associated with community factors, such as the regional deprivation index, as well as personal factors.

Keywords: suicidal ideation, aged, multilevel analysis, socioeconomic factors, Community Health Survey

Volume: 41, Article ID: e2019022
https://doi.org/10.4178/epih.e2019022

Introduction

현대사회에서 노인은 기대 수명의 연장, 핵가족화로 인한 사회적 역할 상실, 대인관계의 약화, 경제적인 불안정, 외로움과 무력감, 지역사회통합 결여 등 복합적인 문제 발생 시 이를 적절하게 대처하지 못하면 자살처럼 극단적이고 부정적인 행동을 취한다는 주장이 있다[1,2]. 또한 독거일 경우, 낮은 교육수준, 사회활동 부재, 우울증상이 높고, 주관적 건강상태와 스트레스가 나쁠수록[3,4,5]. 낮은 사회적지지[6] 등을 자살생각의 원인요인으로 언급되었다. 선행 연구에서는[3.5] 독거노인을 대상으로 개인의 정신건강 문제에 초점을 맞춰 자살 원인을 예측하는 경우가 많으며 노인 자살 생각의 원인적 특성을 개인적 측면과 사회적 측면으로 따로 분리해서 제시되곤 하였다. 또한 노인을 대상으로 사회적 요인을 포함하여 지역적 요인과 비교하는 경우가 드물며, 개인 특성 이외에도 지역적 요인을 포함한 다수준분석을 이용한 국내 논문은 적은 편이다[7,8].

개인건강에 영향을 미치는 요소는 개인 특성뿐 아니라 거주지 주민의 지역적 요인이 복합적으로 작용하며[9] 이러한 측면에서 지역의 사회·물리적 환경이 행정구역 단위로 지역 구분을 연구자의 임의에 맞춰 지역개념으로 활용되고 있다[10]. 관찰 자료단위가 상위집단에 내재되어 있는 자료를 다층적 속성으로 추정하고 그 추정치의 통계적 유의함을 알 수 있는 방법인 다수준분석을 통해 자살생각에 영향을 미치는 개인요인과 지역요인을 파악할 수 있다[11]. 이 연구의 구체적인 목적은 60세 이상 연령의 개인 요인에 따른 자살 생각에 영향을 미치는 요인과 지역요인을 모두 반영하여 자살 생각에 영향을 미치는 요인을 파악하는 것이다.

Volume: 41, Article ID: e2019022
https://doi.org/10.4178/epih.e2019022

Methods

Study subjects

노인복지법에서는 65세 이상을 노인으로 규정하고 있지만 국민연금법 상 노령연금 수급기준이 60세 이상이며, 노인 관련 복지시설의 이용 연령도 동일하다. 본 연구에서 지역사회 활동과 경제적 측면을 고려하여 노인 연령층을 60세로 규정하였다. 2013년 지역사회건강조사 응답 대상자로 총 228,781명이었으며, 이 중 60세 미만을 제외한 총 80,360명에서 필수 문항 15개(연령, 가구유형, 교육수준, 친척(가족) 연락빈도, 친구 연락빈도, 종교활동, 친목활동, 여가활동, 이웃간 신뢰, 안전수준, 월 가구 평균소득액, 경제활동 여부, 우울감 경험, 주관적 스트레스, 주관적 건강수준) 를 응답 거부, 모름으로 대답한 결측 대상자 2,953명을 제외한 77,407명을 최종 선정하였다.

Data

지역사회건강조사는 전국 253개 시·군·구의 만 19세 이상 성인대상으로 보건소 당 평균 900명을 표본 크기로 선정한 자료이며[12], 2013년 원 자료를 이용하였다. 자료 선정한 이유로는 ‘자살예방 및 생명존중문화 조성을 위한 법률’이 2012년에 시행 되었고, 보건복지부는 5년마다 자살예방기본계획을 수립 되었는데 ‘자살예방 및 생명존중문화 조성을 위한 법률’이 시행된 직후와 제2차 자살예방종합대책이(2009-2013년) 끝나는 시점인 2013년에서 노인의 자살 생각에 영향을 미치는 요인이 어떠한 것이 있는지 살펴보기 위함이다.

종속변수는 자살 생각은 최근 1년 동안의 자살생각여부로 구분하였고 “예”로 응답한 자를 자살생각군으로 분류하였다. 독립변수는 연령(60대, 70대, 80대 이상), 가구 유형(노인독거가구, 기타독거가구(노인 가구원과 비혈연가구원 또는 노인가구원과 친인척가구원), 노인부부가구, 자녀동거가구로, 교육수준(무학, 초등학교 졸업, 중학교 졸업, 고등학교 졸업, 대학교 졸업)으로 나눴다. 친척(가족)과 친구의 연락 빈도(한 달에 1번 미만, 한 달에 1-3번, 일주일에 1-3번, 일주일 4번 이상), 종교 활동, 친목 활동과 여가·레저 활동의 여부(한 달에 한번(1회)이상 참여)와 이웃 간의 신뢰, 안전수준의 만족 여부로, 월 가구 평균소득액 수준(200만원 미만, 200-299만원, 300-399만원, 400-499만원, 500만원 이상), 경제활동(일주일 동안 1시간 이상의 수입 활동 또는 18시간 이상 무급가족종사자 활동)로 구분하였다. 우울감 경험의 여부(1년 동안 2주 이상)와 주관적 스트레스(대단히 많

Volume: 41, Article ID: e2019022
https://doi.org/10.4178/epih.e2019022

이 느낌, 많이 느끼는 편, 조금 느끼는 편, 거의 느끼지 않음), 주관적 건강수준(좋음, 나쁨)로, 총 15개의 필수문항으로 개인변수를 구성하였다. 지역변수에서 지역규모는 2013년 행정구역 253개를 체계에 따라 광역시 69개 지역을 대도시로, 시 101개 지역은 중소도시로, 그리고 군 지역인 83개 지역은 농어촌 지역으로 분류하였다. 도시지역과 농촌지역간의 거주 지역에 따라 자살 생각에 영향을 줄 수 있는 지역특성으로 간주하고[13], 지역변수 외 교란요인들의 효과를 최소한으로 줄이기 위하여 행정구역상 3개의 지역규모(대도시, 중소도시, 농어촌지역)로 변인을 구성하였다[8]. 행정구역 변경을 고려하여 2013년 여주군은 여주시로 승격되어 명칭을 변경하였고 연기군은 2012년 군 지역 전체가 세종특별자치시로 통합되어 분석하였다. 지역박탈지수는 지역 간 건강 및 삶의 질의 격차를 밝히는데 사용되는 주요 지표로, 2010년 인구센서스를 9개 기준으로 자동차 미 소유(도시지역, 농촌지역 구분), 열악한 주거환경, 혼자 사는 가구율, 여성 가구주 가구율, 아파트 가구율, 35-64세 중 고등학교 졸업 미만 인구율(하위 교육수준), 하위 사회계층, 노인인구률(65세 이상) 총 9개 지역특성을 Z-점수로 표준화한 수치화이다[14]. 재정자주도는 자주재원 [(지방세+세외수입)/지방단체예산규모]×100을 기준으로 산출되었고 이는 지방정부의 자립적인 지출 능력을 확인할 수 있는 지표이다[8,15]. 기초생활보장수급자비율은 2013년 기초생활보장 수급권자를 기준으로 2010년 인구센서스를 활용하여 지역 내 전체인구 대비 기초생활보장수급자로 100을 곱한 비율로 산출하여 해당 지역 내 전체 인구의 절대적 빈곤상태를 예측할 수 있다[7,8].

Statistical Analysis

2013년 지역사회건강조사 자료를 바탕으로 노인의 자살생각 여부에 따른 개인특성을 기술통계와 χ^2-test로 분석하였고, 노인의 자살생각 가능성과 관련된 개인의 행태와 지역 또는 집단에서 영향력을 파악하기 위한 다수준 분석을 실시하였다.

다수준 분석에서 모형 3개를 구성하였다. Model 1은 기본모형으로, 개인요인과 지역요인이 포함되지 않은 자살생각의 지역 간 차이를 보여주며, Model 2는 개인수준모형으로, 개인요인만을 이용하여 개인특성에 따른 자살생각에 대한 영향을 보았다. Model 3은 개인요인과 지역요인을 포함하여 자살생각에 대한 지역의 영향력을 추정하는 집단수준모형으로 구분하였다. 노인의 자살생각의 전체분포에서 개인의 효과(%)와 지역의 효과(%)를 알아보기 위한 집단 내 상관계수(Intraclass

Volume: 41, Article ID: e2019022
https://doi.org/10.4178/epih.e2019022

correlation coefficient;이하 ICC)를 활용하였으며 Lee & Heo[16]의 방법을 통한 계산식은 아래와 같다.

$$ICC = \frac{\widehat{\sigma_1^2}}{\widehat{\sigma_1^2} + \pi^2/3}$$

σ_i^2: residual variance between communities

$\pi^2/3$: residual variance between individuals

자료 분석은 SAS 9.4 분석기능을 활용하였고 통계적 유의 수준은 α=0.05를 기준으로 하였다.

Ethics

본 연구에서 고려대학교 기관심의위원회 승인을 받았고(IRB. KU-IRB-17-EX-92-A-1) IRB에 의하여 심의 면제를 받았다.

Results

<Table 1>에서 지역사회건강조사 응답자 77,407명 중 11,236(14.5%)명이 죽고 싶다는 생각을 최근 1년 동안 한적이 있다고 응답하였다. 자살생각 응답자 비율은 연령대가 높았고, 친구와 연락빈도에서도 한 달에 1회미만일 때(20.4%), 종교활동(15.2%), 친목활동(19.2%), 여가활동(15.6%)을 하지 않을 때, 이웃간의 신뢰가 불만족할 때(19.5%), 우울감 경험이 있을 때(61.8%) 주관적 스트레스가 높을수록, 주관적 건강수준이 나쁠 때(23.3%) 높았다.

.

<Table 1>

<Table 2>에서 지역의 경제적 수준의 특성을 제시하였다. 지역박탈지수는 가장 낮은 박탈 상태는 -17.7, 가장 높은 박탈 상태는 17.0의 분포를 가지면 평균값 0을 보였다. 지역내의 기초생활보장수

Volume: 41, Article ID: e2019022
https://doi.org/10.4178/epih.e2019022

급자비율은 평균 3.7%으로, 가장 낮은 지역은 0.65%, 높은 지역은 9.6%의 수급자 비율을 보였다. 재정자주도의 평균은 46.4%이며 재원 활용 능력이 낮은 지역은 7.3%이고 재원활용도가 높은 지역은 90%로 나타났다.

<Table 2>

<Table 3>에서 노인의 자살생각에 영향을 미치는 개인요인과 지역요인을 고려한 다수준분석 모형을 제시하였다. Model 1에서는 관련된 독립변수를 제외한 자살생각과 지역 간 차이를 파악하기 위한 것으로, ICC 값이 0.066 산출하였다. 이는 다른 지역에 거주하면서 발생되는 분산의 크기가 전체 자살생각의 분산의 6.6%을 차지하고 뜻을 내포한다.

Model 2의 개인요인을 반영한 결과로 연령이 높을수록, 독거일 때, 친구와 연락 빈도가 한 달에 1회 미만일 때 자살생각 발생가능성이 높았다. 여가활동을 하지 않을 때, 이웃 간의 신뢰에 불만족할 때 자살생각 오즈비가 높았다. 우울감 경험이 있을 때, 주관적 스트레스가 높을수록, 주관적 건강수준이 나쁠 때에 자살생각 경험 가능성이 높았다. Model 2에서 ICC 값이 7.8%로, 노인 자살생각 가능성에 대한 총 분산 중 개인 요인 분산이 차지하는 비율로 해석된다.

Model 3은 개인요인 통제 하에 지역요인이 자살생각에 미치는 영향을 확인하였고 지역요인의 결과는 기초생활보장수급자비율은 노인 자살생각의 오즈비에서 정효과를 보이나 통계적으로 유의하지 않았다. 재정자주도 동일한 결과를 보였다. 지역박탈지수는 1단위씩 증가할 때마다 노인 자살생각의 발생확률이 0.98배 높았고(95% CI, 0.96~0.99) 지역규모는 농어촌지역이 대도시에 비해 자살생각 오즈비가 1.44배 높았다(95% CI, 0.98~2.10). Model 3에서는 노인 자살생각 가능성에 대한 총 분산 중 지역적 요인의 분산이 차지하고 있는 비율이 7.7%로 나타났다.

Volume: 41, Article ID: e2019022
https://doi.org/10.4178/epih.e2019022

<Table 3>

Discussion

개인요인의 연령은 노인 연령대가 높을수록 자살생각 가능성이 높은 결과를 보였는데[17] 과거에 비해 현재에는 고령화와 기대수명 증가로 인해 노인 연령 구간의 생애과정은 명확히 다르기 때문에 연령구간별로 살펴보는 것은 중요한 의미이다. 본 연구에서 친구와 연락빈도에서만 자살생각 경험가능성에 관한 유의미한 결과를 보이는 것은 친척(가족)에 비해 친구와의 연락에서 정서적 지지가 더 크게 작용한다고 보았다[18]. 즉, 공통적인 특성을 가진 사회 구성원과 노인의 상호적 교류는 정서적 공감하거나 교감을 형성하는 요인으로 작용함을 유추할 수 있다. Lee[19]연구에서 사회참여활동은 사회적 고립과 자살생각에서 매개역할을 하는 주된 요소로 사회적 고립이 자살생각에 미치는 영향을 억제할 수 있다고 주장하였으며 본 연구에서도 사회참여활동에 속하는 종교·친목·여가 활동이 하나의 통제요인으로 작용 된 것으로 예측된다. Noguchi et al[17] 연구에서는 신뢰할만한 공동체에 거주하는 노인들은 자기인식과는 상관없이 심리적 고통을 동반한 자살생각 가능성이 줄어 들었으며 본 연구도 인간관계구조에서 신뢰감을 통한 유대감 형성이 정신건강에 긍정적 도움이 된다는 의미로 동일한 결과를 보였다. 다만 추후 이웃간 관계의 강도와 크기를 적용한 영향력을 세밀하게 측정할 수 있는 사회연결망 자료가 요구된다. 우울감 경험이 있을 때, 주관적 스트레스가 높을수록, 주관적 건강수준이 나쁠수록, 자살생각의 가능성이 높았으며 선행연구들과 동일한 결과를 보였다[17,20,21]. 이 요인들은 자살생각에 직접적인 영향을 미치며 특히 자살예방과 관련된 정책에 있어서 중요한 개인요인들로 보여진다.

따라서 노인의 개인요인에 따른 자살생각 발생가능성을 살펴볼 때 자살생각에 영향을 미치는 개인요인은 한가지의 원인이 아닌 여러 복합적인 요인이 서로 영향을 미치고 있음을 확인 할 수

Volume: 41, Article ID: e2019022
https://doi.org/10.4178/epih.e2019022

있다.

본 연구는 선행연구[8]를 토대로 사회경제적 특성을 지닌 지역박탈지수, 재정자주도, 기초생활보장수급자 비율 값을 검토하여 다수준분석에 활용할 수 있는 지역요인으로 적용하였다. 실질적으로 다수준 분석의 설정의 타당성을 추정하기 위해 사회과학 연구에서 ICC값은 5-25%수준이 보편적으로 나타나며 ICC값 5%이하일 경우 단일수준의 모형이 적합하다고 간주할 때[22], Model 3의 ICC값이 7.7%로 다수준 분석에 따른 모형 사용의 적합함을 예측할 수 있다. Model 2의 ICC값 (7.8%)이 Model 1의 ICC값 (6.6%)보다 근소하게 증가하였는데, 국내자료를 이용하여 다수준분석을 수행한 다른 연구들에서도 유사한 결과가 보고된 바 있다[23-25]. 그러나 이들 연구에서도 ICC가 증가한 원인을 제시하지 못하였고, 이를 설명할 수 있는 추가적인 연구가 필요한 상황이다. 다만 지역요인을 고려한 Model 3은 Model 2보다 아주 작으나마 ICC가 감소하였고(0.001), 지역요인으로 고려한 지역박탈지수는 통계적으로 유의하여 유효한 지역요인이라고 해석하는 것이 가능하다고 판단하였다. 그러나 결과적으로 이 모형에서 지역요인(지역박탈지수)이 전체적인 변인을 설명하는 데 기여하는 바는 작다고 판단되며, 이것이 우리나라 지역자료의 특성에 의한 것인지(제공되는 지역특성의 자료가 시군구 단위로 커서 각 개인의 생활환경이나 지역특성을 잘 반영하지 못하는 점 등) 또는 생태학적 오류 등 기타 다른 요인에 의한 것인지 등은 확인하기 어려웠다. 이와 관련되어 추가적인 연구가 필요하다.

Skapinakis et al[26]의 연구는 정신건강에서 지역박탈지수는 정(+)의 방향으로 주장되어 왔으나 Shin & Shin[8]의 연구는 상반된 결과를 보였다. 그 차이를 설명하기 어려운 농어촌 지역의 특성으로 언급하였고 특히 고려되지 않는 개인변수의 효과가 사회적 지지와 지역의 구조적 요인 등의 영향으로 나타난 결과로 보았는데 본 연구의 Model 3에서도 동일한 결과를 보였다. 이러한 지역박탈지수는 연속변수로 절대값을 가지나 OR의 해석을 위해서 차후에 적절한 카테고리로 나누고 분석하는 것이 필요하다. 본 연구에서 추가적으로 지역박탈지수의 결과가 자체적인 변수의 성향인지 다른 변수의 매개의 효과인지를 확인하기 위한 방안으로 기초생활보장수급비율과 재정자주도를 각각 투입하여 산출한 결과에서 Model 3 결과와 유사하게 지역박탈지수는 여전히 역방향으로 나왔고 기초생활보장수급비율과 재정자주도는 통계적으로 유의미하지 않았다. 다만 Model 3에서는 농어촌 지역에서 대도시에 비해 자살생각 오즈비가 1.44배 높게 나왔지만, 유의수준 5% 근접한 결과로(p=0.061) 신

Volume: 41, Article ID: e2019022
https://doi.org/10.4178/epih.e2019022

뢰구간이 1을 포함하고 있다(95% CI, 0.98~2.10). 이는 통계적 검정기준으로 해석하는 한정된 결과로 이해하는 것이 아닌 근사적 신뢰구간을 고려한 자살생각에 영향력이 있는 범위로 해석하는 것이 필요할 것이다.

지역사회건강조사의 자살 관련 문항은 매우 민감한 사항으로 응답자의 반응이 다를 수 있어 지역사회에 적합한 최소한의 문항으로 구성되고[27] Bertolote JM et al[28]의 자살생각 문항을 근거로 사용되고 있다. 또한 4년주기의 순환조사체계로 적용되어 지역보건법의 지역보건의료계획 수립시 활용되어 왔다[29]. 이를 근거로 2013년 자살생각문항 또한 지역의 정신건강 수요를 반영하는데 충분한 자료로 활용이 가능하며 특히 보건의료사업에 지표 자료로 활용됨을 시사한다고 본다.

본 연구의 몇 가지 한계점을 제시하면 첫째, 지역사회건강조사의 자살생각에 관한 문항은 순환주기 구성과 범주형 형태 등으로 제한적 정보로만 이루어졌다. 보완하기 위한 방안으로 국가차원의 자살예방에 맞는 문항구성과 연도별 자료의 활성화가 필요하겠다. 둘째, 지역규모가 외국과는 다르게 한국은 지역주민의 생활권과 활동범위의 경계선을 뚜렷하지 않기 때문에 전국의 지역구분을 행정구역이 아닌 생활권, 공동체 등의 실제 유사한 권역으로 구분할 수 있는 객관적 자료나 선행연구가 요구된다.

결론적으로 본 연구는 60세이상의 성인에서 자살 생각에 개인적 요인 뿐 아니라 지역박탈지수와 같은 지역 요인이 관여한다는 점을 시사한다.

Volume: 41, Article ID: e2019022
https://doi.org/10.4178/epih.e2019022

References

1. Im MY, Kim YJ. A Phenomenological Study of Suicide Attempts in Elders. J Korean Acad Nurs 2011;41(1):61-71.

2. Uncapher H, Gallagher-Thompson D, Osgood NJ, Bongar B. Hopelessness and Suicidal Ideation in Older Adults. Gerontologist 1998;38(1):62-70.

3. Lee SJ, Jeong GI. Meta-analysis of Suicidal Ideation in Elderly. J Korean Data Anal Soc 2016;18(2):1023-1033 (Korean).

4. Yen YC, Yang MJ, Yang MS, Lung FW, Shih CH, Hahn CY, et al. Suicidal ideation and associated factors among community-dwelling elders in Taiwan. Psychiatry Clin Neurosci 2005;59(4):365-371.

5. Lee JE, Lyu JY. Suicide among the Elderly in Korea: A Meta-Analysis. J Korean Gerontol Soc 2017;37(3):601-616 (Korean).

6. Handley TE, Hiles SA, Inder KJ, Kay-Lambkin FJ, Kelly BJ, Lewin TJ, et al. Predictors of Suicidal Ideation in Older People: A Decision Tree Analysis. Am J Geriatr Psychiatry 2014;22(11):1325-1335.

7. Choi KS. A Multilevel Analysis on the Association between Socioeconomic Factors of Region and Residents' Health. The Korean Association for Local Government & Administration studies 2016;30(3):403-426 (Korean).

8. Shin SS, Shin YS. A Multilevel Analysis of Influential Factors on Suicidal Ideation. Journal of Critical Social Welfare 2014;45:230-266 (Korean).

9. Robert SA. Community-level socioeconomic status effects on adult health. J Health Soc Behav. 1998;39(1):18-37.

10. Kim YH, Cho YT. Impact of Area Characteristics on the Health of Vulnerable Populations in Seoul. Korea Journal of Population Studies 2008;31(1):5-30(Korean).

11. Kang SJ. Multilevel models. Seoul: hakjisa; 2016, p.111-118(Korean).

12. Community Health Survey. selection of sample [cited 2017 Jan 10]. Available from: https://chs.cdc.go.kr/chs/index.do(Korean).

13. Mun YH, Im MY. Suicidal Ideation and Associated Factors of the Elderly According to Residence Area- Focusing on the Comparisons between Urban and Rural Areas. Journal of Korean Public Health Nursing 2013;27(3):551(Korean).

14. Yoon TH. Regional health inequalities in Korea: status and policy tasks. Journal of Critical Social Welfare 2010;30:49-77(Korean).

15. Korean Statistical Information Service. Financial Autonomy of Local Governments [cited 2013 Dec 31]. Available from: http://kosis.kr/statHtml/statHtml.do?orgId=101&tblId=DT_ 1YL20891&conn_ path=I2(Korean).

16. Lee JH, Heo TY. A study of effect on the smoking status using multilevel logistic model. Korean J Appl Stat 2014;27(1):89-102(Korean).

17. Noguchi M, Kobayashi T, Iwase T, Suzuki E, Kawachi I, Takao S. Social Capital and Suicidal Ideation in Community-Dwelling Older Residents: A Multilevel Analysis of 10,094 Subjects in Japan. Am J Geriatr Psychiatry 2017;25(1):37-47

18. Woo JW, Woo JM. The Influence of Accepting Attitude toward One's Own Death on Life Satisfaction: the Mediating Effects of Social Support. The Korean Society for Medical Ethics 2016;19(2):225-243(Korean).

19. Lee MS. Does the Social Activity of the Elderly Mediate the Relationship between Social Isolation and Suicidal Ideation? Mental Health and Social Work 2012;40(3):231-259(Korean).

20. Han SS, Yoo Wk, Kang SW, Phee YG. A Study of the Determinants of Suicidal Ideation among the Elderly in Korea. Health and Social Welfare Review 2009;29(1):192-212(Korean).

21. Kim MJ. Suicidal Ideation of the Elderly Using the Data from The 8th Korean Welfare Penal Study JKDAS 2014;16(5):2725-2737.

22. Lee HY, Noh SC. High class statistic analysis-theory and practice. Paju:Beommusa; 2012, p.443-448 (Korean).

23. Song TM, Lee JY. A Multilevel Model Analysis on the Determinants of Smoking Cessation Success Rates. Korean Journal of Health Education and Promotion 2013;30(1):53-64(Korean).

24. Shin SS, Shin YJ. A Multilevel Analysis of an Association between Urbanization and Suicidal Ideation of Adult Korean: Using the 2013 Korea Community Health Survey. Journal of Critical Social Policy 2017;55:187-218(Korean).

25. Song TM, An Anderson Model Approach to the Mediation Effect of Stress-Vulnerability Factors on the Use of Outpatient Care by the Elderly. Health and Social Welfare Review 2013;33(1):547-576(Korean).

26. Skapinakis P, Lewis G, Araya R, Jones K, Williams G. Mental health inequalities in Wales, UK: Multi–level investigation of the effect of area deprivation. Br J Psychiatry 2005;186(5):417-422.

27. Korea Center for Disease Control and Prevention. Development of rotation survey method for Community Health Survey. 2010;55(1):74-83 (Korean).

28. Bertolote JM, Fleischmann A, De Leo D, Bolhari J, Botega N, De Silva D, et al. Suicide attempts, plans, and ideation in culturally diverse sites: the WHO SUPRE-MISS community survey. Psychol Med 2005;35(10):1457-1465.

29. Kim YT, Choi BY, Lee KO, Kim Ho, Chun JH, Kim SY, et al. Overview of Korean Community Health Survey 2012;55(1):74-83(Korean).
